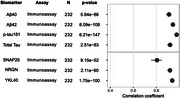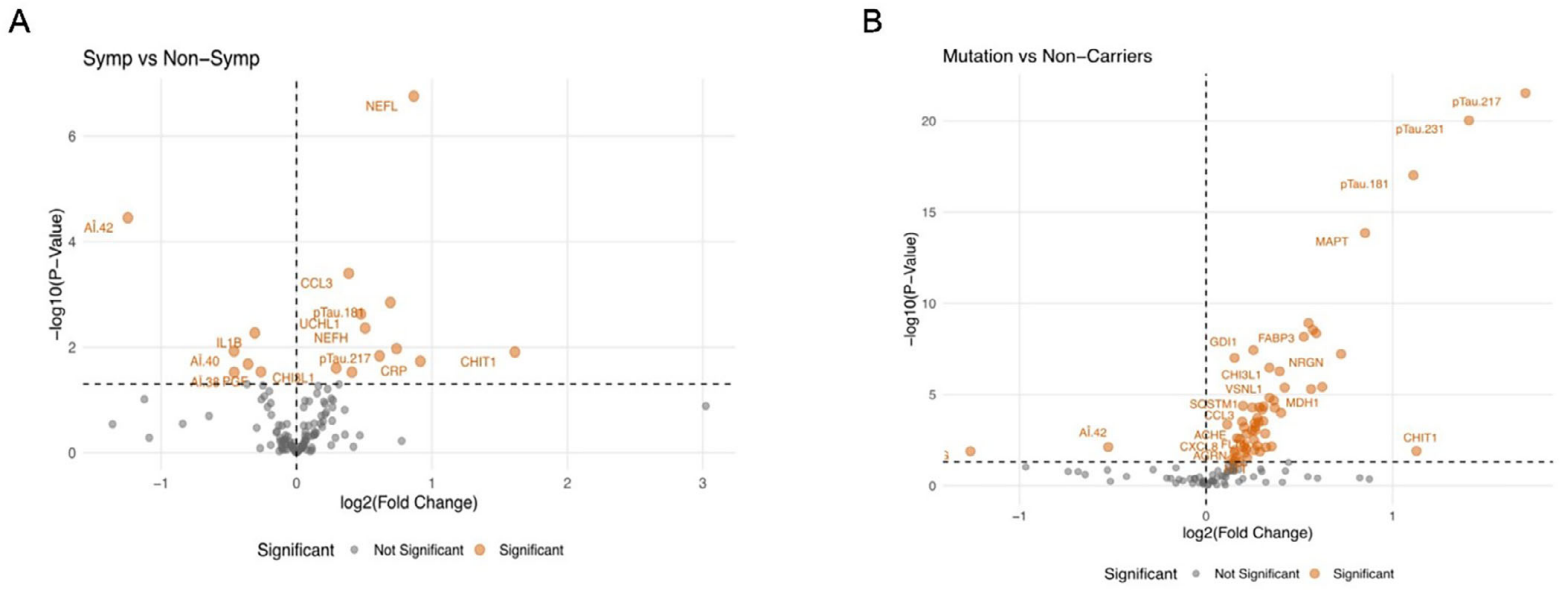# Leveraging Cutting‐Edge NULISA Technology for Proteomic Profiling and Biomarker Discovery in Autosomal Dominant Alzheimer's Disease

**DOI:** 10.1002/alz70856_099818

**Published:** 2025-12-24

**Authors:** Aleksandra Beric, Wenjing Lin, Gina Jerome, Matthew Minton, Sam Preminger, Jennifer Stauber, Bryce Baker, Jason J. Hassenstab, Chengjie Xiong, Brian A. Gordon, Randall J. Bateman, Eric McDade, Jorge J. Llibre‐Guerra, Celeste M. Karch, Carlos Cruchaga, Charlene Supnet‐Bell, Laura Ibanez

**Affiliations:** ^1^ Washington University in Saint Louis, Saint Louis, MO, USA; ^2^ NeuroGenomics and Informatics Center, Washington University School of Medicine, St. Louis, MO, USA; ^3^ Washington University In St. Louis, St. Louis, MO, USA; ^4^ Washington University in Saint louis, Saint Louis, MO, USA; ^5^ Washington University in St. Louis, St. Louis, MO, USA; ^6^ Washington University School of Medicine, Saint Louis, MO, USA; ^7^ Washington University School of Medicine, St. Louis, MO, USA; ^8^ Washington University in St. Louis School of Medicine, St. Louis, MO, USA; ^9^ Washington University in St. Louis, School of Medicine, St. Louis, MO, USA; ^10^ Washington University School of Medicine in St. Louis, St. Louis, MO, USA; ^11^ Hope Center for Neurological Disorders, St. Louis, MO, USA; ^12^ Department of Neurology, Washington University School of Medicine, St. Louis, MO, USA; ^13^ Department of Psychiatry, Washington University School of Medicine, St. Louis, MO, USA

## Abstract

**Background:**

Early disease detection, preceding the onset of symptoms, continues to be one of the biggest challenges in AD research. Individuals that carry AD‐causing mutations provide the perfect opportunity to characterize the natural course of proteomic changes while the new NULISA CNS panel lays out the technical tool to explore AD and inflammatory relevant proteins in a multiplex manner with minimal volume for the first time in Autosomal Dominant AD (ADAD).

**Method:**

We assessed a cross‐sectional set of 232 CSF samples from DIAN participants with NULISA CNS panel from Alamar Biosciences. We are currently assessing the historical collection of DIAN that will be available at the time of the meeting. Then we: (1) validated the NULISA assay by assessing intra and inter assay variability along with established Lumipulse and ELISA assays using Spearman correlation analyses, and (2) identified differentially abundant proteins in mutation carriers compared to non‐carriers, and in symptomatic carriers compared to non‐symptomatic carriers using generalized linear models adjusted by sex and estimated years to onset.

**Result:**

We observed significant (*p* ≤9.15×10^‐52^) strong (ρ>0.80) correlations between measurements obtained with both immunoassay and NULISA for all assayed established (Aβ40, Aβ42, pTau‐181 and total Tau) and emerging (SNAP25, NRGN, YKL40) biomarkers (Figure 1). We identified 17 significantly differentially abundant proteins when comparing symptomatic to non‐symptomatic mutation carriers (Figure 2A), and 59 in the comparison of mutation carriers and non‐carriers (Figure 2B), twelve of them overlapping in both analyses and including the established protein biomarkers. These proteins were enriched in cell death (*p* = 6.8×10^‐4^), glial cell activation (*p* = 6.1×10^‐5^), as well as response to amyloid‐β (*p* = 2.7×10^‐4^).

**Conclusion:**

We have showed that NULISA platform produces reliable measurements, that are highly correlated to those obtained with established methods. Additionally, we demonstrated that utilizing this platform we replicated the known biomarkers, validating the use of NULISA assay in ADAD and DIAN. Additionally, we have identified new proteins associated with presence of AD‐causal mutations or presence of AD symptoms. We are currently investigating trajectories of these proteins in the ADAD disease progression, as well as evaluating their potential as biomarkers.